# Off-axis representation of hyperbolic mirror shapes for X-ray beamlines

**DOI:** 10.1107/S1600577523001492

**Published:** 2023-03-10

**Authors:** Kenneth A. Goldberg, Manuel Sanchez del Rio

**Affiliations:** a Advanced Light Source, Lawrence Berkeley National Laboratory, 1 Cyclotron Road, Berkeley, CA 94720, USA; b ESRF, 71 Avenue des Martyrs, 38000 Grenoble, France; Tohoku University, Japan

**Keywords:** X-ray, mirror, hyperbolic, Wolter, beamline

## Abstract

Mirror-centered, closed-form expressions for hyperbolic surfaces used in X-ray beamlines have been derived. Hyperbolic mirrors create a virtual focus or source point and can be used to lengthen or shorten the effective focal distance of a compound optical system. The derivations here express off-axis segments of a hyperbolic surface in terms of the real and virtual focal distances and the incident glancing angle at the center of the mirror. Conventional mathematical expressions of hyperbolic shapes describe the surfaces in Cartesian or polar coordinates centered on an axis of symmetry, necessitating cumbersome rotation and translation to mirror-centered coordinates. The representation presented here, with zero slope and the origin at the central point, is most convenient for modeling, metrology, aberration correction, and general surface analysis of off-axis configurations. The direct derivation avoids the need for nested coordinate transforms. A series expansion provides a helpful approximation; the coefficients of the implicit equation are also provided.

## Abstract 2

The derivation of the closed-form representation of off-axis hyperbolic and hyperboloidal mirror shapes in a mirror-centered coordinate system for design, modeling, fabrication, and testing is presented. A single equation describes the shape in terms of the conjugate distances and the central angle of incidence.

## 1. Introduction

Since the discovery of X-rays by Wilhelm Röntgen in 1895 (Röntgen, 1895 ▸), scientists have sought ever-improving ways to harness, focus, and control them. Optical systems based on curved X-ray mirrors achieve high reflectivity and efficiency using ultra-smooth surfaces and glancing angles of incidence (Wolter, 1952*a* ▸; Aspnes & Kelso, 1982 ▸; Peatman, 1997 ▸). Many different designs have been explored and tested.

In 1946, Kirkpatrick and Baez demonstrated an X-ray microscope using orthogonally arranged, plane-elliptical focusing mirrors (Kirkpatrick & Baez, 1948 ▸). This configuration is highly effective for point-to-point focusing, yet the field of view is limited by substantial off-axis aberrations (Aspnes & Kelso, 1982 ▸; Lider, 2019 ▸).

Shortly thereafter, in 1952, Wolter proposed a superior X-ray telescope design using hyperbolic mirrors in combination with other shapes to produce X-ray images with a large field of view (Wolter, 1952*a* ▸,*b* ▸). Wolter-type telescope designs use nested coaxial shells and annular pupil shapes (Giacconi *et al.*, 1965 ▸; Mangus & Underwood, 1969 ▸; Weisskopf *et al.*, 2002 ▸). Numerous authors have further advanced these designs while remaining close to the original concept (Zocchi & Vernani, 2007 ▸; Saha & Zhang, 2019 ▸).

Unlike Wolter telescopes, mirrors for synchrotron and free-electron laser beamlines are commonly used in an off-axis configuration with an unobscured pupil that contains the central ray. Early spherical and toroidal shapes, which could be produced at high quality and could be bent to approximate other profiles, have given way to ideal shapes with conic-section profiles. With fabrication and metrology advancing in tandem, arbitrary surface shapes of modest curvature (local radii exceeding hundreds of meters) can be made to nanometre-scale tolerances, and interest in the use of hyperbolic shapes on X-ray beamlines has been renewed (Kodama *et al.*, 1996 ▸). Applications include nano-focusing (Matsuyama *et al.*, 2011 ▸, 2015 ▸) and spectrometers (Chuang *et al.*, 2016 ▸).

Surfaces of revolution, starting from the three canonical conic sections – ellipses, parabolas, and hyperbolas – play important roles in optics. When the two-dimensional conic sections are revolved about a generating axis that passes through the foci (parallel to the direction of collimation in the parabolic case), the results are three-dimensional ellipsoidal, paraboloidal, and hyperboloidal shapes, respectively. Ellipsoidal mirror surfaces focus light ideally from a point source to a point image. Paraboloidal surfaces can either collimate a diverging beam or focus a collimated beam. The reflection of a diverging beam originating from one focus of a hyperboloidal surface creates a virtual source from the other focus. This can be designed either with the mirror–image distance larger than the source–mirror distance, when a concave surface is used, or shorter when a convex surface is used.

**Figure 1:**
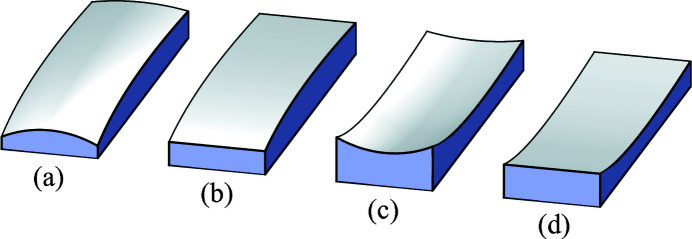
Off-axis hyperboloidal and plane-hyperbolic mirror shapes: (*a*, *c*) hyper­boloids; (*b*, *d*) plane-hyperbolas with no sagittal curvature. Here, the convex and concave shapes are complementary. All have zero slope at the center point.

The addition of hyperboloidal or plane-hyperbolic mirrors to optical systems based on parabolic or elliptical shapes gives flexibility for extending or reducing the effective length of a beamline or telescope. Examples of such shapes are shown in Fig. 1 ▸. Furthermore, with an extended field of view, the sensitivity to errors in position or the incidence angle can be reduced. The use of two optical surfaces is a requisite for optics in imaging applications with a large field of view. Such systems must satisfy or approximate the Abbe sine condition.

In this work, we derive a new representation of 2D plane-hyperbolic and 3D hyperboloidal mirror surfaces using a *vertex* coordinate system rooted at the central point of intersection on a physical mirror. This mirror-centered system has two axes tangent to the surface at this central point, reducing the overall slope across the shape. The third axis is normal to the surface and its coordinate describes the mirror’s height or sag. The description is parameterized by the two conjugate distances and the central angle of incidence. Previous analyses of planar hyperbolic shapes derived only the tangential shape profile or followed a different approach based on nested coordinate transforms (Rah *et al.*, 1997 ▸; Yashchuk *et al.*, 2019 ▸).

This mirror-centered coordinate derivation is complementary to previously published works on ellipsoidal (Goldberg, 2022*a* ▸) and paraboloidal (Goldberg, 2022*b* ▸) mirrors.

## 2. Describing the hyperbola

**Figure 2:**
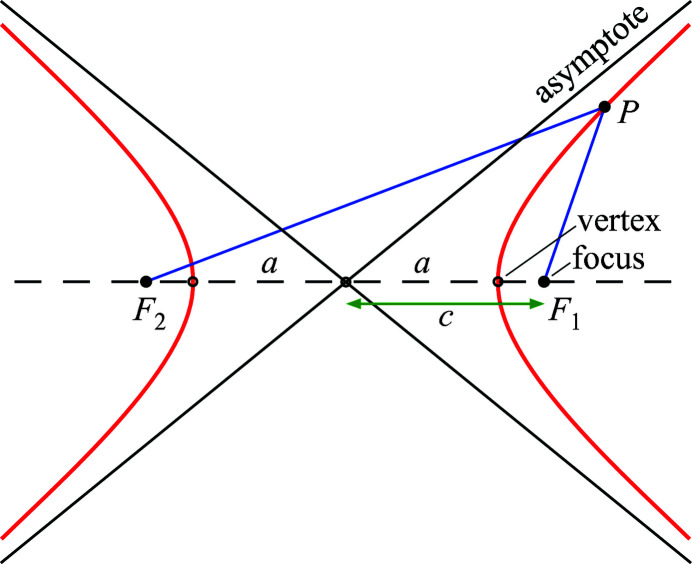
Elements of a hyperbola and definitions of terms: *a* = semi-major axis; *c* = linear eccentricity; *c*/*a* = eccentricity.

As a conic section, a plane-hyperbolic surface can be described in a straightforward way using quadratic polynomials in a two-dimensional plane. Simple and familiar mathematical forms occur with a coordinate system centered on the two mirror-symmetric branches of the hyperbola, as shown in Fig. 2 ▸. The elements of the shape are identified in the figure. We note that the distance between the vertices is 2*a*, the major axis, and half the distance between the foci is *c*, the linear eccentricity. The eccentricity is defined as *e* = *c*/*a*; for hyperbolic shapes, *c* > *a* and *e* > 1. The hyperboloid surface is generated by rotation about the axis connecting *F*_1_ and *F*_2_.

In our analysis, the point *P* in Fig. 2 ▸ will become the center of a mirror that subtends a segment of the hyperbolic shape (a plane-hyperbola or hyperboloid.) The distances from the foci to point *P* will be called *p* and *q*, respectively. Furthermore, the central angles of incidence will be θ, described below.

Regarding Fig. 2 ▸, a plane-hyperbolic surface is isotropic in the direction out of the page. The hyperboloid surface of interest here is generated by rotating about the major axis (the dashed horizontal line). This is sometimes called ‘a hyper­boloid of two sheets’. An alternate hyperboloid surface ‘of one sheet’ can be generated by rotating about the vertical axis of symmetry, creating a single surface like the neck of an hourglass. Such shapes are less important to optical applications.

### 2.1. Defining hyperbola parameters

**Figure 3:**
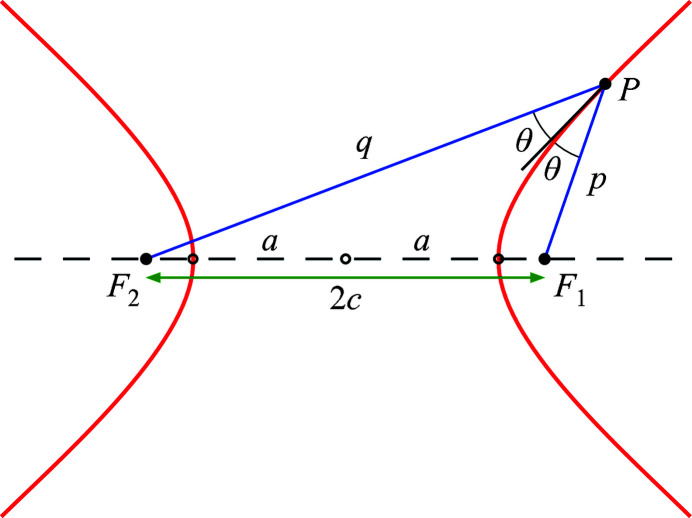
For all points on the surface of the hyperbola or hyperboloid, the difference between the distances to the foci is 2*a* [equation (1)]. The essential optical property of the hyperbola is that the tangent bisects the angle created by the lines to the foci.

Our mathematical solution relies upon one essential property of the hyperbola, shown in Fig. 3 ▸. For any point *P* on the hyperbola or hyperboloid surface, the difference between the distances from that point to the two foci is 2*a*, 

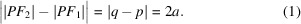

The essential *optical* property of the hyperbola, for our purposes, arises from the fact that these two rays make the same angle with respect to the surface tangent, on opposite sides of the surface (see Fig. 3 ▸). That is, the tangent bisects the angle between the lines to the foci (Wikipedia, 2022 ▸). This property allows the reflected light to appear to emanate from the second focus as a virtual source or image point.

From the Law of Cosines, the square of the linear eccentricity, in terms of *p*, *q*, and θ, is 

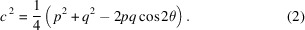

The semi-minor axis, *b*, is defined from *a* and *c* as *b* ^2^ = *c* ^2^ − *a* ^2^. Therefore, from equations (1) and (2),

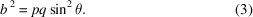




### 2.2. Conventional mathematical description

A simple mathematical description of a hyperbola in the *xy*-plane is 

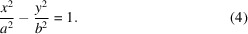

Expanding the shape to three dimensions with rotation about the *x*-axis, the hyperboloidal surface has circular cross-sections in the *yz* planes. With *r* ^2^ = *y* ^2^ + *z* ^2^, now in cylindrical coordinates {*x*, *r*, ϕ}, we have 

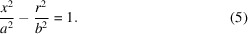

Solving for *r*(*x*), 

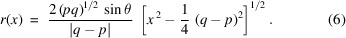

Outside of the gap between the vertices, real solutions exists where 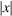 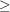 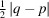 = 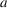 .

## 3. Direct analytic solution of the mirror-centered hyperbolic surfaces

Equation (1) provides the mathematical expression we need to define the surface in the mirror-centered coordinate system, given a known central incidence angle, θ.

**Figure 4:**
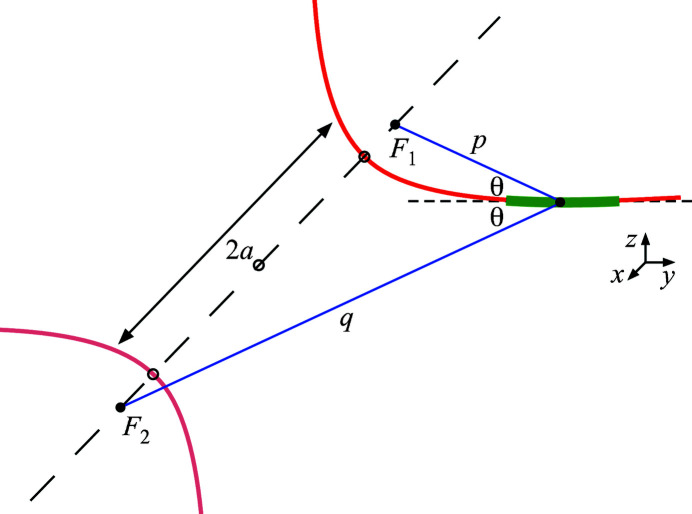
A hyperboloidal surface segment (green curve) is rotated to be tangent to the *xy* plane at the central point of intersection (red dot). The distances to the foci are *p* and *q* and rays from the foci to the mirror center make equal angles θ.

Consider the surface shown in Fig. 4 ▸. One branch of the parent hyperbola has been rotated and translated to center a finite mirror segment (green line) on the origin, with the surface tangent to the *xy* plane. As drawn, the distances from the foci to the mirror center are *p* and *q*, respectively. In (*x*, *y*, *z*) coordinates, as shown, the focal positions are 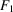 = 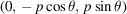 and 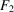 = 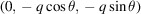 .

The solution we seek has the form *z*(*x*, *y*), with *z*(0, 0) = 0 and d*z*/d*x* = d*z*/d*y* = 0 at the origin. With *q* ≠ *p*, equation (1) can be written as 

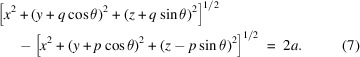

We will separate the *q* > *p* and *q* < *p* cases below. The solution follows by separating the square roots, squaring both sides, isolating the remaining square root, and squaring again. Many terms cancel, leaving a structure that can be solved by quadratic equation,

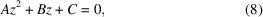




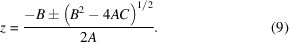

The terms *A*, *B*, and *C* are 

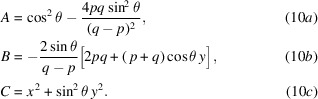

The full solution of the hyperboloid with optical parameters {*p*, *q*, θ} is 

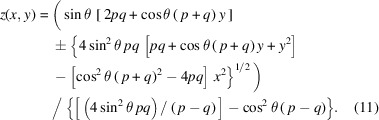

In the plane-hyperbolic case, where the shape is isotropic in *x*, equation (11) reduces to 

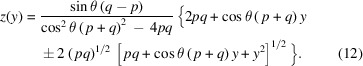

Equations (11) and (12) can be simplified with the substitution of 2*a* = *q* − *p*, and by defining 2*d* = *p* + *q*. For the full hyperboloid, 

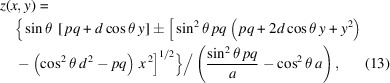

and, for the plane-hyperbola, 

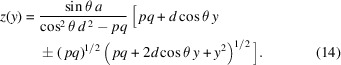

In equations (11) to (14), we use the positive root when *p* > *q*, and the negative root when *p* < *q*.

## 4. Series expansion

Many authors have used polynomial series expansions about the central point of intersection to describe X-ray mirror surface shapes (Howells, 1980 ▸; Peatman, 1997 ▸; Rah *et al.*, 1997 ▸; McKinney *et al.*, 2011 ▸; Yashchuk *et al.*, 2019 ▸); however, such descriptions of hyperbolic and hyperboloidal surfaces are more difficult to find. This mathematical approach simplifies our understanding of central curvatures and can provide an approximation to surface shapes when closed-form representations are not available. Series expansions have also been used to facilitate solutions for mechanically bent mirror substrates, connecting the shape description to beam-bending equations (Rah *et al.*, 1997 ▸; Howells *et al.*, 2000 ▸; Zhang *et al.*, 2010 ▸; Yashchuk *et al.*, 2018 ▸).

A conventional Maclauren series expansion in orders of *x* and *y* takes the form 

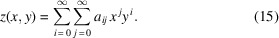

The series expansion of equation (11) was calculated with *Mathematica* (Wolfram Research, 2019 ▸), simplified, and tested empirically. The coefficients up to fouth order, (*i* + *j*) ≤ 4, are listed in equations (17). The surface has zero height at the coordinate origin (*a*_00_ = 0). With the surface tangent to the *xy* plane at that point, the first-order (slope) terms (*a*_10_ and *a*_01_) are also zero. Symmetry about the meridional (*yz*) plane dictates that odd-ordered terms in *x* must also be zero.

It is helpful to define a parameter that behaves like the paraxial focal length, *f*, which appears as a factor in each coefficient, 

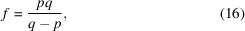




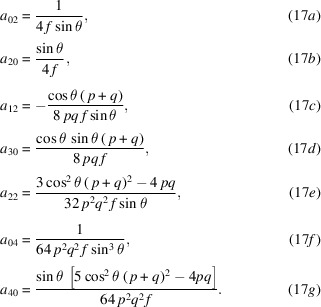

For approximating plane-hyperbolic surfaces with no sagittal curvature, set *a*_*ij*_ coefficients with *j* > 0 to zero.

With the effective paraxial focal length, *f*, the *a*_02_ and *a*_20_ coefficients of *x* ^2^ and *y* ^2^ in equations (17*a*) and (17*b*) match the form of Coddington’s equations (Kingslake, 1994 ▸). The sagittal and meridional radii of curvature are half of the reciprocals of these coefficients, 

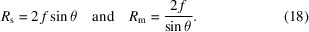

Furthermore, since the mirror’s center point is arbitrary on the parent hyperboloid, we know that locally *R*_m_(*y*) will match this form as the distances to the foci and the angle, *p*, *q*, and θ, vary along the mirror surface.

## 5. Implicit equation

It may be useful in modeling and other contexts to express equation (8) as a polynomial series, 

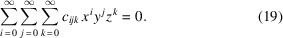

As a conic section, the description of the hyperboloid is limited to quadratic terms. Therefore *c*_*ijk*_ = 0 if *i* + *j* + *k* > 2, giving the expanded expression

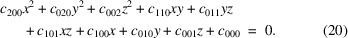

This equation is equivalent to equation (8), and therefore 

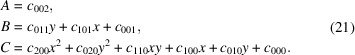

Comparing with equation (10), we obtain the expression of the coefficients of the implicit equation,

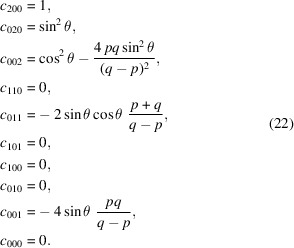




## 6. Conclusion

Hyperbolic and hyperboloidal shapes, which are widely used in X-ray optics applications, including space telescopes, are now being adopted more commonly as optical elements on synchrotron beamlines. Such mirrors are used off-axis, with an unobstructed pupil, preserving the central ray. Optics can be plane-hyperbolic, or they can be considered sections of a rotationally symmetric parent hyperboloid.

We have derived closed-form expressions for plane-hyperbolic and hyperboloidal surfaces based on three optical parameters — the object distance, image distance, and glancing angle of incidence, {*p*, *q*, θ}. The hyperbolic shapes are represented in a mirror-centered coordinate system with zero central slope making the description most conducive to metrology, modeling, and testing. The expressions here can be used to support the creation of compound-optical systems including Wolter-type designs.
